# Arts, health & wellbeing: reflections on a national seminar series and building a UK research network[Fn FN0001]

**DOI:** 10.1080/17533015.2016.1166142

**Published:** 2016-05-10

**Authors:** Theo Stickley, Hester Parr, Sarah Atkinson, Norma Daykin, Stephen Clift, Tia De Nora, Sue Hacking, Paul M Camic, Tim Joss, Mike White, Susan J Hogan

**Affiliations:** ^a^ Faculty of Medicine & Health Sciences, University of Nottingham, Nottingham, UK; ^b^ School of Geographical and Earth Sciences, University of Glasgow, Glasgow, UK; ^c^ Department of Geography, Durham University, Durham, UK; ^d^ Centre for Arts as Wellbeing, University of Winchester, Winchester, UK; ^e^ Sidney De Haan Research Centre for Arts and Health, Canterbury Christ Church University, Folkestone, UK; ^f^ SocArts Research Group, University of Exeter, Exeter, UK; ^g^ School of Nursing and Caring Sciences, University of Central Lancashire, Preston, UK; ^h^ Applied Psychology, Canterbury Christ Church University, Tunbridge Wells, UK; ^i^ Aesop, Brize Norton, UK; ^j^ Centre for Medical Humanities, Durham University, Durham, UK; ^k^ College of Health and Social Care, University of Derby, Derby, UK

**Keywords:** Arts, health, wellbeing, research, network

## Abstract

An account is provided of a UK national seminar series on Arts, Health and Wellbeing funded by the Economic and Social Research Council during 2012–13. Four seminars were organised addressing current issues and challenges facing the field. Details of the programme and its outputs are available online. A central concern of the seminar programme was to provide a foundation for creating a UK national network for researchers in the field to help promote evidence-based policy and practice. With funding from Lankelly Chase Foundation, and the support of the Royal Society for Public Health, a Special interest Group for Arts, Health and Wellbeing was launched in 2015.

## Introduction

Research on arts, health and wellbeing is an established and dynamic field of interest in the UK. This is reflected in the emergence of several university centres of inquiry which are represented through the authorship of this paper.^1^ The growing strength of the field internationally and in the UK, is demonstrated by the fact that it now supports two dedicated peer-reviewed journals: *Arts & Health: An International Journal for Research, Policy and Practice* ^2^ and *The Journal of Applied Arts and Health* ^3^ *.* A further marker of the maturing of this field is the publication of the *Oxford Textbook for Creative Arts, Health and Wellbeing* (Clift and Camic, 2015). There is a need, however, to reach out to the wider academy and to consider the links between arts, health and wellbeing and the more established fields of creative arts therapies^4^ (Hogan, 2015a; Hogan & Coulter, 2014) and medical/health humanities^5^ (Atkinson, Evans, Woods, & Kearns, 2015; Crawford, Brown, Baker, Tischler, & Abrams, 2015; Whitehead, Woods, Atkinson, Macnaughton, & Richards, 2016). The seminar series described here was funded by the UK Economic and Social Research Council (ESRC), and it sought to facilitate wider awareness and networking by drawing together researchers and stakeholders most engaged with arts, health and wellbeing research across the UK. The current state of research, theories, methodologies and applications were shared and discussed.

The series comprised four seminars delivered in Nottingham, Bristol, Glasgow and London during the period March 2012–September 2013. An archive of the series content can be found on the project website.^6^ It was planned by a steering group which comprised ten academics from eight universities as well as an independent consultant from what was then the Public Engagement Foundation^7^ (all are listed as authors of this report). The steering group members reflected a range of disciplines including: arts practice, arts therapy, human geography, nursing, public health, social policy, psychology, social work and sociology. Audiovisual recordings of the following presentations are available on the seminar website:

Seminar 1: Nottingham

• Theo Stickley: Introduction to the seminar series.
• Clive Parkinson: State of the arts?
• Norma Daykin: Arts and health research: Current developments and future prospects.
• Tia De Nora: Arts, wellbeing and health: Theoretical challenges.

Seminar 2: Bristol

• Sarah Atkinson: Setting the context for arts and health research.
• Ellie Byrne: Photography in qualitative mental health research: What happens when research participants take photographs?
• Lynne Frogett: The place of aesthetics in arts and health research.
• Norma Daykin, Jane Wills and Karen Grey: Supporting methodology in arts and health: Results from a Knowledge Transfer Partnership.

Seminar 3: Glasgow

• Alison Phipps: Certainty and doubt: Knowing arts and health from the edge of language.
• Gary Ansdell: Practicing Goethe’s “delicate empiricism” in music therapy research: Finding value and saving the phenomenon.
• Bethan Evans and Charlotte Cooper: Queering arts and health: Engaging with fat activism.
• Christine Borland: Circles of focus: A collaborative visual art project on body donation for creative and artistic research.

Seminar 4: London

• David McDaid: Outcomes and learning from LSE research into economic value: Direct experience from two successful social enterprises.
• Josie Billington and Andrew Jones: Shared reading and chronic pain.
• Gavin Clayton and Susan Potter: Arts on prescription: A study to demonstrate a creative and cost-effective approach to treating mental health.
• Sue Holtum and Val Huet: Complex interventions and RCTs: Learning from the MATISSE study on art therapy with people with a diagnosis of schizophrenia.
• Georgina Charlesworth and Jennifer Wenborn: Group reminiscence for people with dementia and their family careers: Findings from two randomised controlled trials and a qualitative study.

Over the course of the seminar programme, just under 200 academics, postgraduate research students and practitioners from across the UK participated. This paper presents the key outcomes from the seminar series, drawing implications and priorities for the development of arts, health and wellbeing as an academic field of study.

## Context for the seminar series

Over the last thirty years, in the UK, there has been sustained growth of interest in the value of creative arts for health and wellbeing. There is considerable vibrancy in this field, with health trusts, arts organisations and charities working together to promote individual, professional and community health and wellbeing through the arts. Under the leadership of Peter Hewitt, Arts Council England (ACE) encouraged the development of this field. The Department of Health (DH) broadly supported this work and in 2007 various policy documents were published to support it (ACE, 2007; DH/ACE, 2007). Regional networks to support arts, health and wellbeing have been in existence for several years, the most prominent being London Arts and Health Forum, Arts and Health South-West, and West Midlands Arts and these collaborated in the formation of the National Arts and Health Alliance in 2012.^8^

The Royal Society of Public Health (RSPH) has played a substantial role in promoting the value of the arts for health, through its journals, organising and supporting conferences and the annual Arts and Health Awards inaugurated in 2008. In advance of the First International Conference for Culture, Health and Wellbeing in Bristol, 2013^9^ (Coulter, 2013), RSPH also prepared a report on developments in the field since the Windsor Declaration on arts and health in the mid-1990s (Wynn Owen et al., 2013). However, until recently, there was no national research network in the UK focused on arts, health and wellbeing. This seminar group sought to launch such a network to draw together academics from a wide range of disciplines and perspectives in order to share research findings, review methodologies, develop underpinning theories and forge collaborations that will support further development of arts and health research and scholarship.

## Structure and aims of the seminar series

Research on arts, health and wellbeing is found across many academic disciplines and creative agencies, but remains fragmented, and stands in a developing relationship with the creative arts therapies (Hogan, 2015a) and the field of medical or health humanities (Crawford, Brown, Baker, Tischler & Abrams, 2015). The seminars brought into dialogue different academic and practitioner perspectives, involving established researchers and major organisations in the field of arts, health and wellbeing. Through this dialogue, the series sought to meet four aims:

(1) To review the field of arts, health and wellbeing research and debate aims, methods and achievements.
(2) To advance and deepen theoretical, conceptual and methodological developments in researching the role of the arts in health and wellbeing.
(3) to develop strategies for advocacy and knowledge exchange with policy-makers, funders, arts agencies and practitioners.
(4) to establish the foundations for a national network for arts, health and wellbeing research and evidence-based practice.

## The range of academic engagement in arts and health

The first task of the seminar series was to reflect on the range of academic engagement within the field of arts, health and wellbeing. This was addressed throughout the seminar series, with attention to the range of methodologies applied in research (for recent discussions of methods in arts and health research see: Daykin, Attwood, & Willis, 2013; Daykin & Joss, in press; Daykin & Stickley, 2015; Daykin, Willis, Gray, & McCree, 2016; Fancourt & Joss, 2014). A commonly pursued task has been to evaluate arts-based initiatives within primary and secondary care provision for their impacts on health and wellbeing. Examples include arts on prescription in primary care (Bungay & Clift, 2010); arts in mental health care (Secker, Hacking, Spandler, Kent, & Shenton, 2007; Stickley, Hui, & Duncan, 2011); singing for health and wellbeing for people with breathing difficulties (Lord et al., 2012), and hospital-based arts and music (Preti & Welch, 2004; Staricoff, 2006; Staricoff & Clift, 2011). Even the art of magic has been drawn upon in the treatment of hemiplegia in children.^10^

The dominance of this highly programme-focused and applied research within arts and health is not surprising given the requirement for evidence-based practice within health care, but also a de-valuing of the arts and humanities in recent years in the UK. Researchers have endeavoured to build the evidence base for arts, health and wellbeing in terms of the health sector’s identified information needs by producing systematic reviews, evaluation research and quantitative and qualitative studies (Daykin, 2005; Daykin, McClean, & Bunt, 2007; Staricoff, 2006).

Beyond the clinical sector, research has examined the potential value of arts to participants in community settings, particularly in relation to health promotion. Examples include music listening and wellbeing (Batt-Rawden, DeNora, & Ruud, 2005); community dance for health (Stickley, Paul, Crosbie, Watson, & Souter, 2015); community singing for mental health and breathing difficulties (Clift, Manship, & Stephens, 2015; Clift & Morrison, 2011; Morrison et al., 2013), visual arts for mental health recovery (Hacking, Secker, Kent, Shenton, & Spandler, 2006; Parr, 2006; Spandler, Secker, Kent, Hacking, & Shenton, 2007; Stacey & Stickley, 2010; Stickley & Duncan, 2008; Stickley et al., 2011) as well as the benefits of creative arts and music participation for people affected by dementia or Alzheimer’s and their carers (Camic, Williams, & Meeten, 2013; Hara, 2011a, 2011b; Unadkat, Camic, & Vella-Burrows, 2015). The first randomised controlled trial of community singing for a broad cross section of older people (60+) living independently has recently been completed (Coulton, Clift, Skingley, & Rodriguez, 2015; Skingley, Martin, & Clift, 2015). Engagement with visual arts and museum/gallery-based community research with young people, and working-age adults, older adults and those with dementia have all demonstrated benefits (Alcock, Camic, & Barker, 2011; Camic, 2008, 2010; Roberts & Camic, 2011; Solway, Camic, Thomson, & Chatterjee, 2015; Solway, Thomson, Camic, & Chatterjee, 2015; Young, Camic, & Tischler, 2015). Research with new mothers and older women has also explored life transitions and wellbeing though arts engagement (Hogan, 2015b; Hogan, Baker, Cornish, McCloskey, & Watts, 2015; Hogan & Warren, 2012, 2013). These studies often work with expanded definitions of health and wellbeing that encompass a range of personal and social benefits.

Evaluation studies have examined broad impacts including social connectedness or social capital as well as psychosocial impacts such as improved confidence and self-esteem (Ansdell & DeNora, 2012; DeNora, 2011; Spandler et al., 2007). Indirect impacts on wellbeing and ultimately factors known to affect health include social outcomes such as employability (White & Angus, 2003). There is a growing body of research that examines the links between arts and health in relation to wider social concerns such as issues of cultural citizenship (Hogan & Warren, 2013; Parr, 2007). Community arts specifically engage individuals and groups of individuals in arts activities not only for individual gains but more particularly to address both individual disadvantage within a given social context and to strengthen the sense of cohesion and community (White, 2009). There is potential for greater connection between research on public art, public festivals and events, architecture, and design and the field of arts, health and wellbeing. The arts have also been drawn on to address organisational practices and cultures that in turn impact upon health and wellbeing. The best known intervention of this kind is the government-funded Creative Partnerships programme which aimed to impact on learning environments and cultures of practice (Thomson & Sanders, 2010). Whilst ultimately an education and pedagogy intervention, specific programmes often had social and emotional wellbeing as central concerns.

In summary, the seminar series drew together academics and practitioners from across a wide range of research and scholarship, from clinical evaluations through to explorations of arts-based communities of practice. This was challenging, not just because of the breadth and depth of the research base, but also because of the different disciplines and traditions represented. Participants often acknowledged the difficulties in finding a common language to bridge different disciplines and professional practices and to talk about the impacts and challenges of using arts in health and social care. A further challenge was presented by different definitions and understandings of research found in different disciplines. For those most closely aligned with the development of clinical services, evaluation research was a clear priority for the series. However, it was recognised that there are dangers of overlooking other forms of research, not least, the conflation of research with advocacy and the obscuring of more critical theories and perspectives, including those emanating from arts, humanities and social science disciplines.

## Developing appropriate theoretical frameworks

A second challenge for the series organisers was to consider how to best support the development of underlying theory in research on arts and health. Whilst evidence from evaluation research is accumulating, there has been far less attention to developing conceptual and theoretical frameworks for understanding the processes through which the arts may exert their benefits (Camic, 2010; Camic, Baker, & Tischler, 2015; Daykin et al., 2007; Solway et al., 2015). Theoretical perspectives may be constructed from a complex menu of the individual-focused disciplines of psychology, biology or neuroscience (Fancourt, Ockelford, & Belai, 2014; Stickley & Hoare, 2015); the more interactive and structural disciplines of sociology and anthropology (DeNora & Ansdell, 2014); the spatial and temporal disciplines of geography and history (Atkinson & Robson, 2012; Atkinson & Scott, 2015), or more contemplative elements in the humanities including philosophy and studies of the various art forms themselves such as literary studies, media studies and so forth. Whilst within any one of these disciplines, theoretical and conceptual debates exist that may include reference to the arts and which certainly have relevance to arts engagement, there has been little drawing together of these diverse strands into a productive dialogue within the arts and health communities. And there is a particular gap in research beyond the arts therapy traditions examining the nature of arts practice itself, as opposed to the person engaging or the benefits. Kilroy, Garner, Parkinson, Kagan, and Senior (2007) offer a general model of the relationships between the arts and wellbeing in which they propose that a holistic approach to the person interacts with a facilitative environment to generate an openness to change. The arts in this model act as a type of catalyst to change. The seminar series highlighted the need for further work looking at the underlying components of the interventions themselves, to build a more nuanced understanding of arts-based interventions and a stronger theoretical base.

The third seminar, in particular, sought to directly address the need for more theoretical nuance and sophistication in arts and health research to address not only causal relationships, but a deeper understanding of how arts practices function to promote wellbeing (Broderick, 2011). In order to carve out a distinctive intellectual place in an interdisciplinary academy, this seminar challenged researchers to engage further with philosophical reflection on purposes and process, as well as a concern with the quantification of outcomes. The work of critical scholars of arts and health were used to frame this debate in order to challenge delegates:

The specific subject domains of “arts” and “health” do not exist as concrete entities, but are shifting, amorphous and contested, subject to competing knowledge claims …. (Broderick, 2011, p. 95)

The current landscape of social theory is relevant to arts and health researchers, particularly with new thinking around affect and theories of practice (Stewart, 2007). Researchers could usefully engage with this kind of work to secure wider academic engagement across disciplinary boundaries within universities. From this perspective researching the role of the arts for and in health can be seen as a creative conversation between the places, sites and spaces in which it occurs and the radical possibilities of its practice as a critique of contemporary medicalised models of health care.

## Advocacy, policy development and networking

The third aim of the seminar series, to develop strategies for advocacy and knowledge exchange was discussed at each of the seminars as delegates were very keen to engage with the current political agenda. In the course of the project, Lord Howarth, a UK parliamentarian and former Minister for the Arts, challenged the group to develop a manifesto for knowledge exchange and this activity was incorporated into the programme (see below for developments in this respect subsequent to the seminar series).

The final aim, to establish a UK network for arts and health research was fully met as during the programme, funding was acquired from Lankelly Chase Foundation to establish such a network. On an interim basis the steering group for the seminar series acted as a steering group to support the establishment of a new network.

## Moving forward with a national network for arts, health and wellbeing for research and evidence-based practice

Funding from Lankelly Chase Foundation has been of considerable value in helping to maintain the momentum of the ESRC-funded seminar series and build a UK network of researchers working in the field of arts, health and wellbeing.^11^ The RSPH agreed to host the network as a Special Interest Group (SIG) with additional resources in the form of secretarial support and general membership benefits from the Society. The aims of the new RSPH SIG are: sharing current research and best practice; organising conferences, seminars and workshops, and influencing government policy as a professional body. An inaugural meeting of the SIG took place in London on in June 2015 and attracted over 70 delegates. The PhD students and early career researchers who engaged with the seminar series have also created a network on social media and this has 85 members. They have independently organised a follow-up seminar.

An innovative feature of the work of the RSPH SIG for arts, health and wellbeing is the organisation of webinars on arts and health topics. The first of these in November 2015 focused on two UK arts on prescription initiatives run by Arts and Minds in Cambridge and Pathways2Wellbeing a spin-out initiative from the University of Hertfordshire.^12^ A second webinar in February 2016 was devoted entirely to the innovative and world-leading research of Teppo Särkämö and his team at the University of Helsinki on music and stroke recovery and singing and dementia.^13^ Further webinars are planned for April and July 2016, with a further programme of four webinars envisaged for 2016–17. The SIG is also supporting a major research conference on arts and dementia in March 2017.^14^

In terms of political engagement, the Special Interest Group is acting as the research partner for the UK All Party Parliamentary Group (APPG) inquiry on Arts, Health and Wellbeing which will run over two years 2016–17. The secretariat for the APPG is provided by the National Alliance for Arts, Health and Wellbeing, supported by the London Arts and Health Alliance. Management of the APPG secretariat is provided by Alex Coulter, Director of Arts and Health South West. A webpage for the APPG is maintained on the National Alliance website and details of their activities can be found there.^15^

The RSPH SIG for Arts, Health and Wellbeing has made an auspicious start and it represents a very welcome initiative in helping to further establish the place of the creative arts in health care and the promotion of health and wellbeing in the wider context of public health. Membership of the SIG is open to anyone with an active interest in arts and health research and the development of evidence-based practice in this field. Further details of membership can be obtained from RSPH (see endnote 8 below).

## Funding

The Seminar Series was funded by the Economic and Social Research Council [grant reference: ES/J022527/1]. Funding from Lankelly Chase Foundation [grant reference: 10290/13564] has supported the development of a UK national network for arts, health and wellbeing.

## Notes

1. Further significant centres for research on arts, health and wellbeing in the UK not so represented are: Arts for Health, Manchester Metropolitan University: http://www.artsforhealth.org/; Arts and Health Research Group, Manchester Metropolitan University and Manchester School of Art: http://www.art.mmu.ac.uk/research/artsandhealth/; Arts in Health, Queen's University Belfast: http://www.qub.ac.uk/research-centres/ceipe/Projects/ArtsinHealth/; the Centre for Performance Science, Royal College of Music see: http://www.rcm.ac.uk/cps/; Music for Health Research Centre, Anglia Ruskin University: http://www.anglia.ac.uk/arts-law-and-social-sciences/department-of-music-and-performing-arts/our-research/music-for-health-research-centre; Scottish Music and Health Network, University of Edinburgh: http://www.eca.ed.ac.uk/reid-school-of-music/research/projects/scottish-music-and-health-network; the Social and Evolutionary Science Research Group, Oxford University: http://www.psy.ox.ac.uk/research/social-and-evolutionary-neuroscience-research-group-senrg; The Centre for Arts as Wellbeing, University of Winchester: http://www.winchester.ac.uk/AAW.

2. 
*Arts & Health: An International Journal for Research, Policy and Practice* is edited by Profs Paul Camic, Stephen Clift and Norma Daykin: http://www.tandfonline.com/toc/rahe20/current.

3. 
*Applied Arts and Health* is edited by Prof.. Ross Prior, University of Northampton: http://www.intellectbooks.co.uk/journals/view-Journal,id=169/ . For networking associated with the journal see: http://appliedartsandhealth.ning.com/.

4. Three modalities of arts therapy are regulated by the Health Care Professions Council: art therapy, drama therapy and music therapy: http://www.hcpc-uk.co.uk/. Professional associations exist for these three professions: British Association for Music Therapy: www.bamt.org; British Association of Arts Therapists: www.baat.org; British Association of Dramatherapists: www.badth.org. There is also a UK Association for Dance Movement Psychotherapy: http://www.admt.org.uk/.

5. Details of university centres for scholarship and training in the field of Medical Humanities can be found via the Association of Medical Humanities website: http://amh.ac.uk/.

6. For details of outputs from the ESRC Seminar Series on Arts, Health and Wellbeing see: http://www.artsandhealthresearch.ac.uk/artshealthandwellbeing/index.aspx.

7. The Public Engagement Foundation subseqently became Aesop, Arts Enterprise with a Social Purpose. See www.aesop.org.uk for details.

8. For the National Alliance for Arts, Health and Wellbeing see: http://www.artshealthandwellbeing.org.uk/recent-developments/national-alliance-arts-health-and-wellbeing.

9. For an archive of information on this conference, and the forthcoming Second Culture, Health and Wellbeing International Conference in June 2017 see: http://www.culturehealthwellbeing.org.uk/ An excellent report on the conference is also available (Coulter, 2013): http://www.culturehealthwellbeing.org.uk/userfiles/documents/conf/CHW_Conference_Report.pdf.

10. See: http://breatheahr.org/breathe-magic-hemiplegia/ for details of work on magic and hemiplegia at Guy's and St. Thomas' Hospital in London.

11. A further national network established around the same time as the RSPH SIG is the National Alliance for Museums, Health and Wellbeing. For details see: https://museumsandwellbeingalliance.wordpress.com/about/.

12. Arts and Minds Cambridge: http://www.artsandminds.org.uk/; Pathways2Wellbeing: http://www.pathways2wellbeing.com/. Members of the SIG are able to access the webinar in the members area of the SIG pages on the RSPH website: https://www.rsph.org.uk/en/membership/special-interest-groups/arts-and-health/.

13. For detais of Teppo Särkämö's work see: https://tuhat.halvi.helsinki.fi/portal/en/person/sarkamo.

14. Details of all forthcoming events organised through the SIG can be found on the RSPH website (see endnote 7).

15. For the UK All Party Parliamentary Group for Arts, Health and Wellbeing see: http://www.artshealthandwellbeing.org.uk/APPG.
